# Influence of Depression and Sleep Quality on Postoperative Outcomes after Total Hip Arthroplasty: A Prospective Study

**DOI:** 10.3390/jcm11133845

**Published:** 2022-07-02

**Authors:** Umile Giuseppe Longo, Sergio De Salvatore, Alessandra Greco, Martina Marino, Giulia Santamaria, Ilaria Piergentili, Maria Grazia De Marinis, Vincenzo Denaro

**Affiliations:** 1 Research Unit of Orthopaedic and Trauma Surgery, Fondazione Policlinico Universitario Campus Bio-Medico, Via Alvaro del Portillo, 200, 00128 Rome, Italy; s.desalvatore@unicampus.it (S.D.S.); alessandra.greco5@gmail.com (A.G.); martilibia@gmail.com (M.M.); i.piergentili@unicampus.it (I.P.); denaro@policlinicocampus.it (V.D.); 2 Research Unit of Orthopaedic and Trauma Surgery, Department of Medicine and Surgery, Università Campus Bio-Medico di Roma, Via Alvaro del Portillo, 21, 00128 Rome, Italy; 3 Research Unit Nursing Science, Campus Bio-Medico di Roma University, 00128 Rome, Italy; g.santamaria@unicampus.it (G.S.); m.demarinis@unicampus.it (M.G.D.M.)

**Keywords:** depression, sleep, mental status, health quality, Total Hip Arthroplasty, hip replacement, quality of life outcomes

## Abstract

The trend of Total Hip Arthroplasty (THA) is projected to grow. Therefore, it has become imperative to find new measures to improve the outcomes of THA. Several studies have focused attention on the influence of psychological factors and sleep quality on surgical outcomes. The consequences of depressive states may affect outcomes and also interfere with rehabilitation. In addition, sleep quality may be an essential factor in determining surgical outcomes. To our knowledge, few articles focus on the influence of these factors on THA results. The present study investigates a possible correlation between preoperative depression or sleep quality and postoperative outcomes of THA. This study was conducted with 61 consecutive patients undergoing THA from January 2020 to January 2021. Patients were assessed preoperatively using GDS and PSQI, and six months postoperatively using FJS-12, SF-36, WOMAC, PSQI, and GDS. To simplify comparisons, the overall scores were normalized to range from 0 (worst condition) to 100 points (best condition). A total of 37 patients (60.7%) were classified as depressed and 24 as not depressed (39.3 %) in the preoperative assessment. A low–moderate positive correlation between preoperative GDS score and FJS-12 (rho = 0.22, *p* = 0.011), SF-36-PCS (rho = 0.328, *p* = 0.01), and SF-36-MCS (rho = 0.293, *p* = 0.022) scores at six-month follow-up was found. When the normalized preoperative GDS score was high (no depression), the FJS-12, SF-36-PCS, and SF-36-MCS scores tended to increase more compared to the other group. Statistically significant differences between the two groups were found in postoperative FJS-12 (*p* = 0.001), SF-36-PCS (*p* = 0.017), and SF-36-MCS scores (*p* = 0.016). No statistically significant correlation between preoperative PSQI score and postoperative outcome measures was found. Preoperatively depressed patients had a low–moderate positive correlation with postoperative SF-36 and FJS-12 scores. There was no correlation between sleep quality and postoperative outcome measures of THA.

## 1. Introduction

Osteoarthritis is considered the most prevalent joint disease globally [1]. Specifically, hip osteoarthritis is related to significant social limitations and associated disability [2]. As a result, Total Hip Arthroplasty (THA), the treatment of choice for this condition, is performed at considerable rates and constitutes one of the most commonly performed surgical procedures worldwide [3]. In the UK, it is estimated that the number of THA performed each year is 43,500 [3]. Despite the high statistics, this trend is projected to grow in the future, following the increase in diagnoses and demand, due to the progressive ageing of the population and the development of new implants [3,4,5].

In light of such epidemiological data and projections, it has become imperative to find new measures to minimize postoperative complications and improve THA outcomes. However, implant design, prosthesis material, or the surgeon’s skills are not the only factors that could influence postoperative outcomes.

Several studies have recently focused on the influence of psychological factors and sleep disorders on surgical outcomes. Psychosocial status has been identified as a significant contributor to depression and poor outcomes in orthopedic trauma patients [6]. Furthermore, the consequences of depressive status may affect the overall surgical outcomes, also interfering with rehabilitation [7]. As a result, providing an early psychological intervention could positively influence patient outcomes.

In addition to a patient’s psychological condition, sleep quality may also be an important factor in determining surgical outcomes, given its role in memory, learning, and quality of life [8]. Some authors have explored the possible relationship between sleep disturbance and surgical outcomes in orthopedic patients [9,10].

To our knowledge, although numerous studies are now bringing attention to the influence of sleep and depression on the outcome of various surgical procedures, there are few articles focusing on the effects of these factors on THA outcomes.

The present study investigates a possible correlation between preoperative depression or sleep quality and postoperative outcomes of THA.

## 2. Materials and Methods

Patients who underwent THA from January 2020 to January 2021 were assessed preoperatively using the Geriatric Depression Scale (GDS) and the Pittsburgh Sleep Quality Index (PSQI) and six months postoperatively using the Forgotten Joint Score 12 (FJS-12), the 36-Item Short-Form Health Survey (SF-36), the Western Ontario and McMaster Universities Osteoarthritis Index (WOMAC), PSQI, and GDS. Patients with severe hip osteoarthritis (Kellgren–Lawrence Classification Grades III-IV) [11], extreme and chronic pain, who underwent hip replacement surgery, and at least a six-month follow-up postoperatively were included in the study. Informed consent was obtained from all subjects involved in the study.

All patients were treated with the same surgical implants and underwent complete hip arthroplasty (both anterolateral and posterior approaches). During the follow-up, no revisions were made. All the procedures were performed by the same senior surgeon, skilled in hip arthroplasty.

Patients with Kellgren–Lawrence Classification Grades I–II, simultaneous bilateral hip replacement, hip resurfacing, endoprosthesis, revision surgery, and/or patients with cognitive impairment were excluded from the study.

### 2.1. Preoperative Depression

Before surgery, preoperative depression was assessed with the validated Italian form of the GDS survey [12]. This is a 30-item self-scored questionnaire that excludes somatic and psychotic symptoms. Given that items are scored dichotomously, the tool can be used with all patients [12]. The GDS is defined as a self-report questionnaire and requires an average of 20 min to complete [12]. The score ranges from 0 to 30. A score of ≤9 is considered normal, 10–19 indicates mild depression, and 20–30 indicates severe depression [13]. Therefore, patients with a GDS of more than 9 were identified as depressed. To simplify comparisons, the overall score was normalized to range from 0 (worst condition) to 100 points (best condition). Therefore, patients with higher scores were more depressed.

### 2.2. Preoperative Sleep Quality

The PSQI questionnaire validated in the Italian language [14] was used to assess the preoperative sleep quality in patients who underwent THA. The PSQI is a 19-item self-scored questionnaire used to assess the sleep quality of patients [15]. The PSQI total score ranged from 0 (the best quality of sleep) to 21 (worst quality of sleep). A PSQI score less than 5.5 indicates good sleep [8]; therefore, patients with PSQI values lower than 5.5 were considered good sleepers and patients with PSQI values more than 5.5 as bad sleepers. To simplify comparisons, the overall score has been normalized to range from 0 (worst condition) to 100 points (best condition). Therefore, patients with higher scores experienced more sleep disturbance.

### 2.3. Postoperative Scores

The following questionnaires were administered six months postoperatively: FJS-12, SF-36, WOMAC, PSQI, and GDS.

The FJS-12 is an outcome questionnaire designed to evaluate joint awareness [16]. It was translated and validated in the Italian language [17]. This survey consists of 12 questions with a five-point Likert response format that is summed to obtain scores ranging from 12 to 60 [16]. To simplify comparisons, the overall score has been normalized to range from 0 (worst condition) to 100 points (best condition).

The SF-36 score was translated and validated in Italian [18]. This questionnaire is designed to offer general health indicators. The questionnaire comprises 36 questions divided into eight different sections [18]. These eight sections were clustered into two components: the Physical Component Summary (SF-36-PCS) and the Mental Component Summary (SF-36-MCS). In addition, a single unscaled question on health changes in the previous year is also included (health change) [18]. The overall score ranged from 0 (worst condition) to 100 points (best condition).

The WOMAC [19] is a clinical orthopedic score used to assess patients’ pain, stiffness, and physical function. The WOMAC score was translated and validated in Italian [20]. It consists of 24 questions with a zero- to four-point Likert response format that is summed to provide a score between 0 and 96. To simplify comparisons, the overall score has been normalized to range from 0 (worst condition) to 100 points (best condition).

### 2.4. Statistical Analysis

A priori power analysis was performed with SAS OnDemand for Academics by setting the following parameters: a significance level of 0.05, a statistical power of 80%, and a correlation of −0.382 between WOMAC and Preoperative GDS [21]. The minimum total sample size amounted to 51 subjects.

To evaluate preoperative depression, patients were divided into two groups based on their preoperative GDS score, with a threshold of 9 [13]: no depression in patients with a GDS score ≤ 9 and the presence of depression in patients with a GDS score > 9.

To evaluate the preoperative quality of sleep, patients were divided into two groups based on their preoperative PSQI score with a threshold of 5.5 [8]: good sleep quality in patients with PSQI ≤ 5.5 and bad sleep quality in patients with PSQI > 5.5.

The Shapiro–Wilk test was used to assess the normal distribution of the data. Since the data were not normally distributed, the differences in the scores between the groups (depression vs. no depression and good sleep vs. bad sleep) were calculated using the Independent-Samples Mann–Whitney U Test. The correlation between the preoperative scores (GDS and PSQI) and the postoperative scores was calculated using Spearman’s correlation.

SPSS for Windows (version 26; Armonk, NY, USA: IBM Corp) and R Core Team (2020) version 4.0.3 were used to perform all statistical analyses and for the figures.

## 3. Results

Overall, 86 eligible patients were approved to participate and registered in the study; however, only 61 patients (28 women and 33 men) completed the questionnaires at the six-month postoperative follow-up. The average age was 74 ± 10 years.

### 3.1. Preoperative Depression

A total of 37 patients (60.7%) were classified as depressed, whereas 24 were classified as not depressed (39.3 %). The median normalized preoperative GDS score for depressed patients was 46.7 (range: 16.7–66.7), whereas the score for non-depressed patients was 85 (range: 73.3–96.7) (*p* < 0.001).

Correlation of preoperative depression score to postoperative outcome measures revealed a low–moderate positive correlation between normalized preoperative GDS score and FJS-12 (rho = 0.22, *p* = 0.011), physical component summary (SF-36-PCS) (rho = 0.328, *p* = 0.01), and mental component summary (SF-36-MCS) (rho = 0.293, *p* = 0.022) scores at six-month follow-up (Table 1). As normalized preoperative GDS score increased (more depressed patients), the FJS-12, SF-36-PCS, and SF-36-MCS scores also increased (better outcomes).

**Figure 1:**
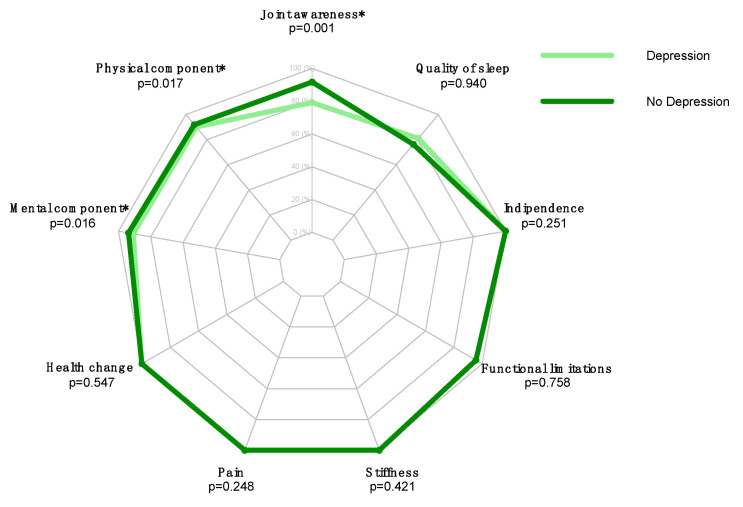
Radar plot preoperatively depressed and not depressed patients assessing FJS-12, SF-36-PCS, and SF-36-MCS scores. * = *p* < 0.05.

Statistically significant differences between the two groups (preoperatively depressed and not depressed patients) were found in postoperative FJS-12 (*p* = 0.001), SF-36-PCS (*p* = 0.017), and SF-36-MCS scores (*p* = 0.016) (Table 2, Figure 1). Patients without depression showed higher scores than depressed patients (Table 2).

### 3.2. Preoperative Quality of Sleep

A total of 52 patients (85.2%) were classified as bad sleepers, whereas 9 were classified as good sleepers (14.8%). The median preoperative PSQI score for patients with bad sleep was 57.1 (range: 19 to 71.4), whereas the score for patients with good sleep was 76.2 (range: 76.2 to 85.7) (*p* < 0.001).

No statistically significant correlation between preoperative PSQI score and postoperative outcome measures was found (Table 3).

**Figure 2:**
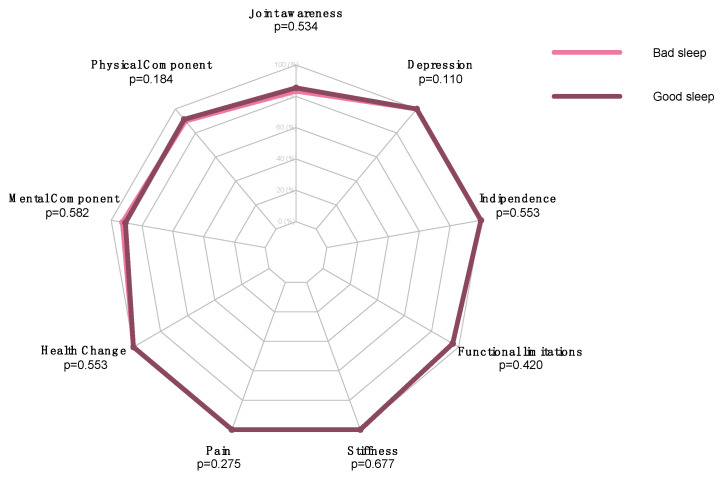
Radar plot assessing differences between bad and good sleepers.

There were no statistically significant differences between the preoperative bad and good sleepers in postoperative outcomes (Table 4, Figure 2).

## 4. Discussion

The main findings of the present study suggest that depression influences outcomes of THA negatively. On the other hand, sleep quality does not seem to influence the postoperative outcomes of patients who underwent THA.

### 4.1. Depression and THA Outcomes

A state of depression was found in 60.7% of patients, a value well above the reported prevalence of depression in the American population, estimated to be 8.4% by the American National Institute of Mental Health. This large discrepancy in values may be due to the utilized score, which was the GDS. In epidemiological studies, this score is targeted at geriatric patients and screens for depression and depressive symptoms. In addition to this, given that the average age of included patients was 74, depression prevalence can be compared to the prevalence of geriatric depression in the outpatient setting, which is estimated to be 27% [22]. Furthermore, the prevalence of depression in orthopedic trauma patients can reach 45%, which hints at a possible correlation between orthopedic patients and depressive symptoms [7]. This is relevant because, according to the results of the present study, a depressive state has been shown to negatively influence THA outcome and recovery, especially when compared to patients without depressive symptoms. Lastly, in some patients, the disability due to hip osteoarthritis could influence the self-reported depression evaluation.

The study by Duivenvoorden et al. agreed with our findings, reporting that preoperative depressive symptoms are related to smaller changes in Hip disability and Osteoarthritis Outcome Score (HOOS) [23]. Moreover, the satisfaction rate was lower in depressed patients compared to controls after 12 months [23].

Data revealed a low–moderate positive correlation between preoperative depression measured through the normalized GDS score and postoperative SF-36 value. More specifically, there was an increase in the scores of both the SF-36-PCS and SF-36-MCS components of the SF-36 questionnaire, which showed a slight improvement in mental and physical condition six months postoperatively. Nguyen et al. suggest that THA may play a role in improving patients’ depressive status, given that mental conditions showed improvement postoperatively [24]. The SF-36 is an extensively validated and accepted method for determining the quality of life in orthopedic patients, and in general, it is expected that SF-36 scores generally improve postoperatively [25,26,27]. Santić et al. evaluated the impact of THA or total knee arthroplasty (TKA) two years postoperatively on the quality of life of elderly patients using the SF-36 score [25,28]. They found significant improvement in all assessment levels, excluding mental health [25]. The authors attributed the lack of improvement in the mental health category to high preoperative motivation of patients that was reflected in an already good mental health condition. However, this claim was not accompanied by scores or values that had recorded preoperative and postoperative anxiety, depression, and other mental conditions. Ng et al. produced a large prospective cohort study with an SF-36 score, reporting that quality of life improved postoperatively and slightly declined after the 18-month mark but remained higher than the preoperative value [26]. These studies evaluated patients’ quality of life postoperatively using the SF-36 score instead of using it as a measure of outcomes of the THA procedure; however, their findings are still relevant to the present study.

The findings of the present study highlight that although both the depressed group (DG) and non-depressed group (NDG) had positive outcomes, the latter group showed more significant improvement in SF-36 scores and, ultimately, better outcomes. Therefore, to improve THA outcomes equally across different patient groups, certain factors must be taken into consideration and managed properly, and one of these factors is mental health condition.

To further highlight the importance of comparing outcomes between groups, it was found that the FJS-12 values improved more in the NDG. Larsson et al. highlight the importance of this score in evaluating implants claimed to provide patient satisfaction, and the clinical performance of implants [29]. Their findings highlight the value of the FJS-12 score as a very reliable tool for understanding the success and quality of implants and inevitably also a tool to evaluate the procedure’s success. Furthermore, an improvement in physical condition potentially demonstrates an excellent surgical outcome despite the preoperative depressive state. The low–moderate positive correlation between preoperative depression and postoperative FJS-12 value supports this hypothesis. A patient with a “forgotten joint” during daily activity usually reported a higher satisfaction value and a lower depression rate [30,31,32]. Despite the postoperative physical and mental improvement identified in depressed patients postoperatively, to truly understand the influence of depression on THA outcomes, it is crucial to evaluate the differences between the DG and NDG. It was found that patients without depression preoperatively, compared to those with depression, had better outcomes in terms of SF-36 and FJS-12 scores.

Seagrave et al. explored the association between depression (diagnosed by a psychiatrist) treated with medication and outcomes of THA one year postoperatively [33]. Anxiety and depression groups experienced a greater improvement in the Oxford Hip Score, while there was a less global joint improvement, and they were more likely to have major complications. The authors concluded that patients should be treated for depressive symptoms pre- and postoperatively to maximize THA outcomes. The results of this study are similar to those of the present study, despite the use of a different categorization approach for mental health conditions [33]. The strength of utilizing psychiatrist-diagnosed patients instead of a self-scored questionnaire such as the GDS would be the precision and accuracy of medical diagnosis. However, it is more likely that such a strict definition excludes patients with undiagnosed or mild depressive symptoms and includes those who already have a well-managed mental illness. Another study written by Götz et al. retrospectively assessed the role of depression in outcomes of THA and TKA after a one-year follow-up, in a cohort of 5447 patients [34]. Preoperatively, a high degree of depression was found in these patients, which agrees with the present findings. The study concluded that patients with anxiety and depression symptoms show significantly worse clinical outcomes [34]. These findings are consistent with the results of the present study; however, their retrospective design is a potential cause of greater confusion and bias compared to the prospective approach. Furthermore, the study by Götz et al. utilized the EQ-5D index to score patients. EQ-5D is a generic preference-based measure of health-related quality of life in contrast to the SF-36, which is non-preference based. Usually, preference-based instruments are utilized for the economic evaluation of cost, where the primary outcome measure is quality of life; instead, non-preference-based instruments focus on a specific condition and or population of interest [35]. As a result, when evaluating outcomes based on a population, the preferred measure would be the SF-36 survey utilized in the present study. These data are supported by the fact that depression has become an independent risk factor for delayed recovery and worsened outcomes for many physical conditions [7].

Tristaino et al. evaluated the effectiveness of psychological support in patients undergoing THA and TKA. In this study, it was suggested that short-term recovery of functionality is attributed to clinical factors. On the contrary, the authors reported that in the long term follow up, full recovery is closely related to the preoperative degree of functionality and postoperative psychological status [36]. Patients receiving psychological support had a more significant improvement in scores of the SF-36-PCS and significantly better SF-36-MCS scores over time postoperatively. In a systematic review, Bay and colleagues reported that only in two of the seven studies included the patients benefits from psychological counselling on THA outcome [37]. In two randomized controlled trials included in the review, the psychological interventions also seem to improve the outcomes of THA in the long term follow up. Psychological counselling, especially for patients with preoperative negative mental health symptoms, may be promising for improving THA outcomes, despite the lack of literature. However, there are few high-quality studies on psychological interventions and THA; therefore, further clinical trials are required to obtain significant results [37].

### 4.2. Sleep and THA Outcomes

A statistically significant correlation was not found between preoperative sleep quality and postoperative outcome measures. Moreover, there was no significant correlation between preoperative bad sleepers and good sleepers and their respective postoperative outcomes. These data suggests that sleep quality is not relevant to THA outcomes. However, more data are needed to evaluate this correlation further.

To our knowledge, there are no previous studies discussing the role of sleep quality on THA outcomes. However, most of the other literature regarding sleep quality and THA focuses on the possible influence of surgery on sleep quality improvement [38,39,40].

A randomized, double-blind, controlled study by Gong et al. aimed to report a correlation between sleep quality and TKA outcomes [41]. Patients were administered zolpidem postoperatively in order to improve their quality of sleep. Findings showed a significantly positive relationship between sleep efficacy and improved functional training in patients with lower analgesic intake. The authors concluded that improved quality of sleep due to short-term zolpidem application improved recovery from TKA [41]. This study is crucial because it provides data suggesting that sleep quality does influence the postoperative outcome of total arthroplasty of weight-bearing joints. Such data further highlight that the influence of sleep quality on outcomes of joint replacement procedures is still misunderstood and undeniably requires more thorough research.

## 5. Limitations

The present study has some limitations. Firstly, this is not a randomized study. Moreover, the use of Patient-Reported Outcome Measures (PROMs) is influenced by the subjectivity of the evaluation. However, although PROMs can be affected by subjectivity, data were collected using a variety of well-researched surveys, which were carefully selected based on their applicability to the current study and their accepted use in clinical research. Postoperative follow-up was performed after 6-months, which allowed for a substantial recovery period postoperatively. Furthermore, there is no indication of a gold standard instrument for evaluating depression and sleep quality. However, results are often dependent on the instruments used to assess these factors and on timing, causing discordance on this topic. sWithal, considering that preoperative depressive state is not confirmed by HR-QOL scores, our conclusion can only be tentative. The present study explores the impact of depression and sleep quality on outcomes of THA, which is a topic that is not yet well researched in the orthopedic field. However, due to the projected increase in THA procedures, it is imperative to be aware of the risk factors that negatively influence THA outcomes. Identifying such risk factors can lead to finding solutions to them and will ultimately benefit patients receiving THA.

## 6. Conclusions

Depressed patients seem to experience a slight improvement in mental and physical outcomes after THA. However, although THA outcomes seem to be positive in depressed patients, some PROMs are better in those who are not depressed. In addition, there was no correlation found between sleep quality and postoperative outcome measures of THA and no statistically significant difference between preoperatively good sleepers and bad sleepers. The lack of a statistical correlation suggests that it would be more worthwhile to manage mental health symptoms instead of preoperatively tackling sleep quality. Further clinical trials are required to better assess preoperative depression and to establish if psychological approaches could influence THA outcomes.

## Figures and Tables

**Table 1:** Correlations between preoperative GDS and postoperative scores.

Parameter	GDS	
	Rho	*p*-Value
FJS-12	0.322	0.011 *
SF-36 PCS	0.328	0.01 *
SF-36 MCS	0.293	0.022 *
SF-36 Health change	0.057	0.665
WOMAC Pain	0.128	0.325
WOMAC Stiffness	0.062	0.632
WOMAC Functional Limitations	0.085	0.513
WOMAC Overall	0.086	0.509
BARTHEL	0.083	0.525
PSQI	−0.014	0.913

FJS-12: Forgotten Joint Score 12; SF-36: 36-item Short-Form Health Survey; SF-36-MCS: 36-item Short-Form Health Survey—Mental Component Summary; PSQI: Pittsburgh Sleep Quality Index; SF-36-PCS: 36-item Short-Form Health Survey—Physical Component Summary; WOMAC: Western Ontario and McMaster University Osteoarthritis Index. * = *p* < 0.05.

**Table 2:** Median (min–max) values of the GDS score.

Parameter	Depression (*n* = 37)		No Depression (*n* = 24)		*p*-Value
	Median	Range	Median	Range	
FJS-12	79.2	22.9–100	91.7	56.3–100	0.001 *
SF-36 PCS	90.0	30–98.8	91.9	75.6–100	0.017 *
SF-36 MCS	90.8	30.5–100	93.8	83.8–100	0.016 *
SF-36 Health change	100.0	75–100	100.0	75–100	0.547
WOMAC Pain	100.0	75–100	100.0	90–100	0.248
WOMAC Stiffness	100.0	87.5–100	100.0	100–100	0.421
WOMAC Functional Limitations	95.6	55.9–100	95.6	79.4–100	0.758
WOMAC Overall	96.9	63.5–100	96.9	83.3–100	0.690
BARTHEL	100.0	50–100	100.0	100–100	0.251
PSQI	81.0	33.3–85.7	76.2	47.6–95.2	0.940

FJS-12: Forgotten Joint Score 12; SF-36: 36-item Short-Form Health Survey; SF-36-MCS: 36-item Short-Form Health Survey—Mental Component Summary; PSQI: Pittsburgh Sleep Quality Index; SF-36-PCS: 36-item Short-Form Health Survey—Physical Component Summary; WOMAC: Western Ontario and McMaster University Osteoarthritis Index. * = *p* < 0.05

**Table 3:** Correlations between preoperative PSQI and postoperative scores.

Parameter	PSQI	
	Rho	*p*-Value
FJS-12	−0.021	0.87
SF-36 PCS	0.127	0.329
SF-36 MCS	0.017	0.9
SF-36 Health change	−0.19	0.143
WOMAC Pain	0.114	0.383
WOMAC Stiffness	0.152	0.243
WOMAC Functional Limitations	−0.049	0.708
WOMAC Overall	−0.03	0.819
BARTHEL	−0.06	0.645
GDS	0.185	0.153

FJS-12: Forgotten Joint Score 12; GDS: Geriatric Depression Scale; SF-36: 36-item Short-Form Health Survey; SF-36-MCS: 36-item Short-Form Health Survey—Mental Component Summary; PSQI: Pittsburgh Sleep Quality Index; SF-36-PCS: 36-item Short-Form Health Survey—Physical Component Summary; WOMAC: Western Ontario and McMaster University Osteoarthritis Index.

**Table 4:** Median (min–max) values of the PSQI score.

Parameter	Bad Sleep (*n* = 52)		Good Sleep (*n* = 9)		*p*-Value
	Median	Range	Median	Range	
FJS-12	83.3	22.9–100	85.4	56.3–100	0.534
SF-36 PCS	90.0	30–100	91.3	86.3–100	0.184
SF-36 MCS	92.7	30.5–100	90.8	83.8–96.8	0.582
SF-36 Health change	100.0	75–100	100.0	75–100	0.553
WOMAC Pain	100.0	75–100	100.0	95–100	0.275
WOMAC Stiffness	100.0	87.5–100	100.0	100–100	0.677
WOMAC Functional Limitations	95.6	55.9–100	95.6	86.8–100	0.420
WOMAC Overall	96.9	63.5–100	96.9	90.6–100	0.419
BARTHEL	100.0	50–100	100.0	100–100	0.553
GDS	100.0	33.3–100	100.0	96.7–100	0.110

FJS-12: Forgotten Joint Score 12; GDS: Geriatric Depression Scale; SF-36: 36-item Short-Form Health Survey; SF-36-MCS: 36-item Short-Form Health Survey—Mental Component Summary; PSQI: Pittsburgh Sleep Quality Index; SF-36-PCS: 36-item Short-Form Health Survey—Physical Component Summary; WOMAC: Western Ontario and McMaster University Osteoarthritis Index.

## Data Availability

The data presented in this study are available on request from the corresponding author.

## Abbreviations

DG	Depressed Group
FJS-12	Forgotten Joint Score 12
GDS	Geriatric Depression Scale
HOOS	Hip disability and Osteoarthritis Outcome Score
NDG	Non-Depressed Group
PSQI	Pittsburgh Sleep Quality Index
SF-36	36-item Short-Form Health Survey
SF-36-MCS	36-item Short-Form Health Survey—Mental Component Summary
SF-36-PCS	36-item Short-Form Health Survey—Physical Component Summary
THA	Total Hip Arthroplasty
TKA	Total Knee Arthroplasty
WOMAC	Western Ontario and McMaster University Osteoarthritis Index
